# Integration of Single-Cell and Bulk Transcriptome to Reveal an Endothelial Transition Signature Predicting Bladder Cancer Prognosis

**DOI:** 10.3390/biology14050486

**Published:** 2025-04-28

**Authors:** Jinyu Yang, Wangxi Wu, Xiaoli Tang

**Affiliations:** 1 Queen Mary School, Nanchang University, Nanchang 330031, China; 4217121019@email.ncu.edu.cn (J.Y.); wangxi.wu@se21.qmul.ac.uk (W.W.); 2 School of Basic Medical Sciences, Nanchang University, Nanchang 330031, China

**Keywords:** endothelial cells, pan-cancer analysis, bladder cancer, prognostic signature

## Abstract

Endothelial cells (ECs) are critical drivers of tumour progression, and their angiogenic process has been widely studied. However, the post-angiogenic transition of tip endothelial cells after sprouting remains insufficiently characterised. In this study, we utilised single-cell RNA sequencing analyses to identify a novel EC transition signature associated with endothelial permeability, migration, metabolism, and vascular maturation. Within the transition pathway, we discovered a critical EC subpopulation, termed tip-to-capillary ECs (TC-ECs), that was enriched in tumour tissues. Comparative analyses of TC-ECs with tip and capillary ECs revealed distinct differences in pathway activity, cellular communication, and transcription factor activity. The EC transition signature demonstrated substantial prognostic significance, validated across multiple cancer cohorts from TCGA data, particularly in bladder cancer. Subsequently, we constructed a robust prognostic model for bladder cancer by integrating the EC transition signature with multiple machine-learning techniques. Compared with 31 existing models across the TCGA-BLCA, GSE32894, GSE32548, and GSE70691 cohorts, our model exhibited superior predictive performance. Stratification analysis identified significant differences between different risk groups regarding pathway activity, cellular infiltration, and therapeutic sensitivity. In conclusion, our comprehensive investigation identified a novel EC transition signature and developed a prognostic model for patient stratification, offering new insights into endothelial heterogeneity, angiogenesis regulation, and precision medicine.

## Simple Summary

Bladder cancer is a common and serious disease, and finding better ways to predict how it will progress is crucial for improving treatment. In this study, we focused on a particular group of cells in the blood vessels, called endothelial cells, which play an important role in tumour growth. These cells change in specific ways as cancer progresses, and understanding these changes can help us predict the development of cancer. By studying these changes in cells from cancer tissue, we developed a prognostic prediction model that can predict the outcome for bladder cancer patients. Our results show that the model can identify patients who are at higher risk to predict prognosis and provide more information to help doctors and patients choose the treatments.

## 1. Introduction

Cancer remains a leading cause of mortality worldwide, with approximately 20 million new cases and nearly 9.7 million deaths reported in 2022 [1]. In the United States alone, it is estimated that 2,041,910 new cases and 618,120 deaths will happen in 2025 [2].

Despite advancements in innovative therapies, the inherent heterogeneity of tumours continues to pose significant challenges, limiting treatment efficacy [3]. Traditional cancer studies focusing on single cancer types often fail to capture the complexity and diversity observed across different malignancies. Pan-cancer studies have thus emerged to overcome these limitations, providing a more comprehensive understanding by identifying common molecular drivers across multiple cancer types [4]. Comparative analyses between primary and metastatic tumours across cancers further aid in revealing shared and unique features, facilitating better prediction of therapeutic outcomes and prognosis [5].

Investigating tumour heterogeneity at the cellular level necessitates high-resolution technologies, notably single-cell RNA sequencing (scRNA-seq), enabling detailed characterisation of the tumour microenvironment (TME) [6]. Within the TME, endothelial cells (ECs) play crucial roles in tumour progression, primarily through their functions in angiogenesis and immune modulation [7]. Based on their anatomical locations, ECs can be categorised into capillary, lymphatic, arterial, and venous ECs [8]. Additionally, during angiogenesis, a specialised EC subpopulation called tip ECs guides the growth of new blood vessels and subsequently matures into stable ECs upon the formation of a new vascular network [9]. Anti-angiogenic therapies targeting EC-driven angiogenesis have demonstrated survival benefits [10]. However, their efficacy varies significantly across different cancer types, likely due to EC heterogeneity within tumours [11]. Although EC heterogeneity is recognised, the precise transition from tip ECs to mature capillary ECs remains inadequately explored, particularly from a pan-cancer perspective.

Consequently, this study aims to bridge this critical gap by integrating published scRNA-seq datasets across multiple cancer types to systematically characterise the transition from tip ECs to capillary ECs and identify a robust endothelial transition signature. Subsequently, bladder cancer was specifically selected for further validation in light of the critical role of EC transition in it. Additionally, we developed a robust prognostic model based on the signature using multiple machine-learning algorithms, providing a novel tool for guiding patient stratification and personalised treatment strategies in bladder cancer.

## 2. Methods

### 2.1. Data Collection

The single-cell data were downloaded from CellxGene, the Curated Cancer Cell Atlas, Tabula Sapiens, and GSE210347, comprising 280 samples from bladder, breast, gastric, colorectal, lung, liver, ovarian, pancreatic, and prostate cancers and corresponding normal tissues (Supplementary Table S1) [12,13]. Only 10x scRNA-seq datasets of human tissues were chosen to rule out possible technology-induced bias. Quality control initially excluded low-quality datasets with fewer than 10,000 detected genes or lacking expression data for mitochondrial genes or key endothelial markers (*PECAM1*, *CDH5*, *VWF*, and *TIE1*). Additionally, the bulk RNA sequencing samples of bladder cancer and associated survival information were obtained from four public datasets, including TCGA-BLCA (*n* = 412), GSE32894 (*n* = 308), GSE32548 (*n* = 131), and GSE70691 (*n* = 49).

### 2.2. Identification of EC Subpopulations

The scRNA-seq data analysis was based on the Seurat R package (v4.1.3) [14]. Samples with fewer than 1000 total cells or fewer than 100 ECs were removed. Cells were further filtered based on the following criteria: total read counts less than 500 or more than 20,000, detected genes fewer than 500 or more than 5000, and mitochondrial gene percentage greater than 10%. Subsequently, all 148,864 ECs were extracted for further dimension reduction and clustering. The expression profiles were log-normalised, and the data were then scaled after identifying the 2000 most variable genes. Principal component analysis (PCA) was conducted, followed by batch effect correction using the Harmony R package (v1.2.0) [15]. Dimensionality reduction was performed using UMAP with 40 dimensions, and clustering was performed at a resolution of 0.1. Some contaminating clusters, showing the signatures of epithelial cells (*KRT19* and *KRT8*), fibroblasts (*COL1A1* and *DCN*), macrophages/monocytes (*MS4A6A*, *S100A8*, and *CSTA*), T cells (*CD3D* and *CD3E*), and smooth muscle cells (*TAGLN* and *MYL9*) were excluded from downstream analysis. Ultimately, five EC subpopulations were then identified, including arterial, vein, capillary, lymphatic, and tip ECs, using marker genes from published studies and the Cell Taxonomy database (Table 1) [16]. Tip ECs and capillary ECs were subsequently extracted and processed through the same workflow, resulting in ten clusters at a resolution of 0.2. Tissue preferences of the ten clusters were revealed by calculating the STARTRAC-dist index (Ro/e) to determine their distribution across different tissues [17].

### 2.3. Investigation of Endothelial Transition from Tip ECs to Capillary ECs

Pseudotime analysis was performed using Monocle3 to investigate potential cellular transitions between tip ECs and capillary ECs [18,19]. The root of the cellular trajectory was defined based on differentiation levels, which were calculated using the CytoTRACE R package (v0.3.3) [20]. Genes exhibiting significant expression changes (false discovery rate < 0.05) along the trajectory were identified and integrated with marker genes from the transitional cluster (T1) to identify potential transition genes involving EC transition in cancer progression. The functions of these transition genes were further investigated through GO enrichment analysis using the clusterProfiler R package (v4.8.3) [21]. The key differential transcription factors in the transitional cluster were estimated by the SCENIC R package (v1.3.1) [22]. The enriched pathways were identified by using gene sets from MSigDB database with the irGSEA R package (v3.2.4) [23].

### 2.4. Exploring the Prognostic Significance of Endothelial Transition Signature

Endothelial transition signature scores were generated by gene set variation analysis (GSVA) using the GSVA R package (v1.50.0) [24]. The correlation between these scores and prognosis was then examined across 33 cancer cohorts from TCGA. For eight cancers showing significant correlations (*p* < 0.05), Kaplan–Meier analysis was performed for further validation with the log-rank test. Additionally, immune and stromal infiltration levels across the 33 TCGA cancers were estimated using the ESTIMATE algorithm to assess their correlation with the signature score [25]. Finally, tumour mutation burden (TMB) and microsatellite instability (MSI) data from the cBioPortal database for these 33 cancers were used to investigate their association with the signature score.

### 2.5. Cellular Communication Analysis

Cellular communication analysis and visualisation were conducted by the CellChat R package (v 2.1.2) with the built-in human-specific ligand–receptor interaction database, including secreted signalling, ECM–receptor, and cell–cell contact pathways [26]. Firstly, the overexpressed ligand–receptor interactions among 15 cell types were identified, and communication probabilities were calculated using the triMean method. To ensure robustness, interactions detected in fewer than 10 cells were filtered out. Communication probabilities at the signalling pathway level were computed by aggregating all related ligand–receptor pairs. Furthermore, network centrality scores were calculated to identify the dominant sender, receiver, mediator, and influencer cell populations within the inferred communication networks.

### 2.6. Construction of Prognostic Model Based on Endothelial Transition Signature

The endothelial transition signature was chosen for prognostic prediction model construction in bladder cancer. The TCGA-BLCA cohort was chosen as the training set to identify relevant features and construct prognostic prediction models using various machine-learning methods, including CoxBoost, Lasso, Ridge regression, elastic net (Enet), generalised boosted regression modelling (GBM), partial least squares regression for Cox (plsRcox), random survival forest (RSF), stepwise Cox, supervised principal components (SuperPC), and survival support vector machine (survival-SVM) [27]. The C-index helped evaluate and determine the best model, which was validated across external datasets, including GSE32894, GSE32548, and GSE70691. The model’s performance was further assessed through ROC curves. The predictive accuracy of the model was also compared with published prognostic models with risk-scoring formulas retrieved from PubMed. Models based on long non-coding RNA were excluded due to their unstable expression, interpretability, and lower presence in public datasets. Ultimately, 31 models were chosen after excluding models with gene mismatch rates exceeding 50% across integrated cohorts (Supplementary Table S2). The protein expression levels of genes involved in the model were validated by comparing immunohistochemistry (IHC) images from the HPA database between the tumour and normal groups.

### 2.7. Characterisation of High-Risk Patients

The distribution of risk scores was evaluated across different T, N, and M stages to assess their correlation with cancer progression in tumour size, lymph node invasion, and distant metastases. Patients were subsequently divided into high-risk and low-risk groups based on an optimised cut-off by the surv_cutpoint function of the survminer R package (v0.4.9) for the signature score. Differentially expressed genes between risk groups were identified using the limma R package (v3.56.2), followed by gene set enrichment analysis (GSEA) with the clusterProfiler R package [21]. To investigate the association between risk scores and immunotherapy response, immunophenoscore (IPS) values from the TCIA database were assessed. Additionally, Tumour Immune Dysfunction and Exclusion (TIDE) scores were used as indicators of potential immunotherapy responsiveness [28]. The difference in clinical drug sensitivity for bladder cancer was demonstrated by the oncoPredict R package (v0.2) to predict chemotherapy efficacy by calculating half-maximal inhibitory concentration (IC50) values [29].

### 2.8. Statistical Analysis and Plot

All statistical analyses were conducted using R Statistical Software (v4.3.1). The Mann–Whitney U test was employed for comparisons of continuous variables, and the Chi-square test was employed for comparisons of categorical variables. A *p*-value or false discovery rate < 0.05 indicated statistical significance. Significance levels are denoted as follows: *p* < 0.05 (*), *p* < 0.01 (**), *p* < 0.001 (***), and *p* < 0.0001 (****).

## 3. Results

### 3.1. Identification of EC Transitional Cluster Associated with Tumours

**Figure 1:**
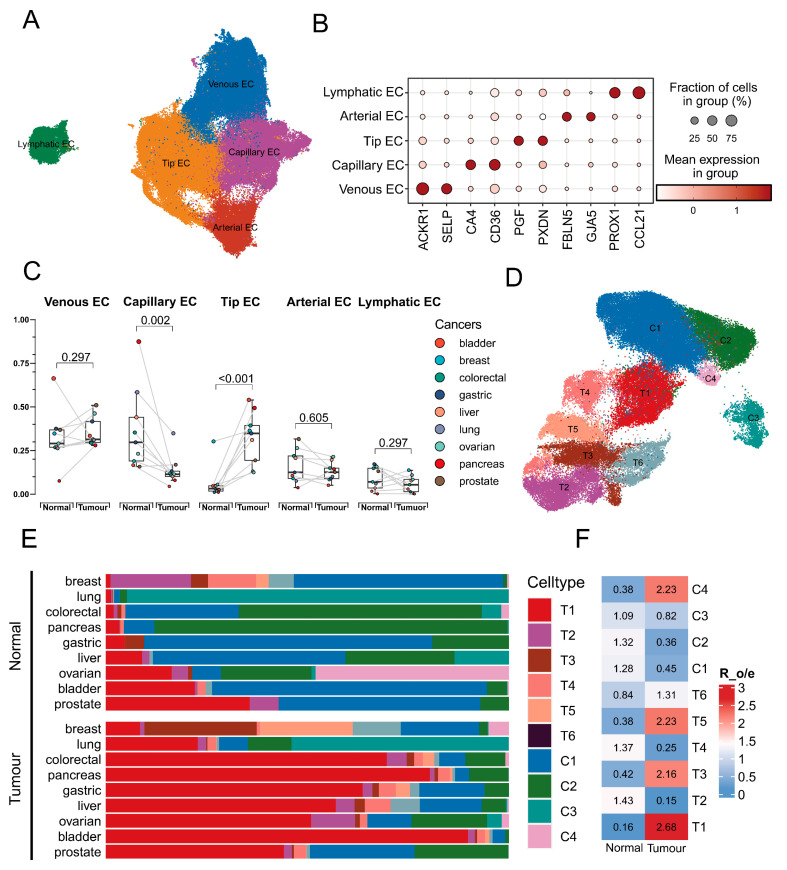
Landscape of subpopulations of endothelial cells. (**A**) UMAP visualisation of the scRNA data identifying 5 distinct endothelial cell subpopulations among 148,864 cells. (**B**) A dot plot shows the expression of marker genes across endothelial cell subpopulations. The colour intensity represents the expression level, while the dot size indicates the proportion of cells expressing the marker. (**C**) The boxplot identifies the significant differences (*p* < 0.01) in the abundance of all cell types between normal and tumour tissue across cancers. Statistical significance was assessed by the Wilcoxon rank-sum test, with lines indicating paired comparisons. (**D**) UMAP visualisation of capillary endothelial cell subpopulations and tip endothelial cell subpopulations identifies 10 cell types. (**E**) A bar diagram visualises the cellular proportion of the 10 identified cell types across 9 cancers, where colour denotes distinct cell types and bar length represents the cellular proportion. (**F**) A heat map shows the enrichment level of distinct subpopulations of tip endothelial cells and capillary endothelial cells between normal and tumour tissues.

After stringent quality control, 148,864 ECs from normal and tumour tissues across nine cancer types were retained for analysis. UMAP visualisation revealed the single-cell landscape of ECs, identifying five major clusters at a resolution of 0.1 (Figure 1A). The dot plot demonstrated that these cell types were well distinguished by respective conventional markers (Figure 1B). A paired Wilcoxon rank-sum test indicated significant differences (*p* < 0.01) in the proportions of tip ECs and capillary ECs between normal and tumour tissues (Figure 1C). Subsequently, tip ECs and capillary ECs were subdivided into ten clusters to explore potential transitional states (Figure 1D). Notably, the abundance of cluster T1, identified as a potential transitional population, was obviously elevated and enriched in tumour tissues (Figure 1E,F). This cluster was thus designated as “tip-to-capillary endothelial cells (TC-ECs)”, which was the focus of further analysis.

### 3.2. Identification of the Transition Signature from Tip Cells to Capillary Cells

**Figure 2:**
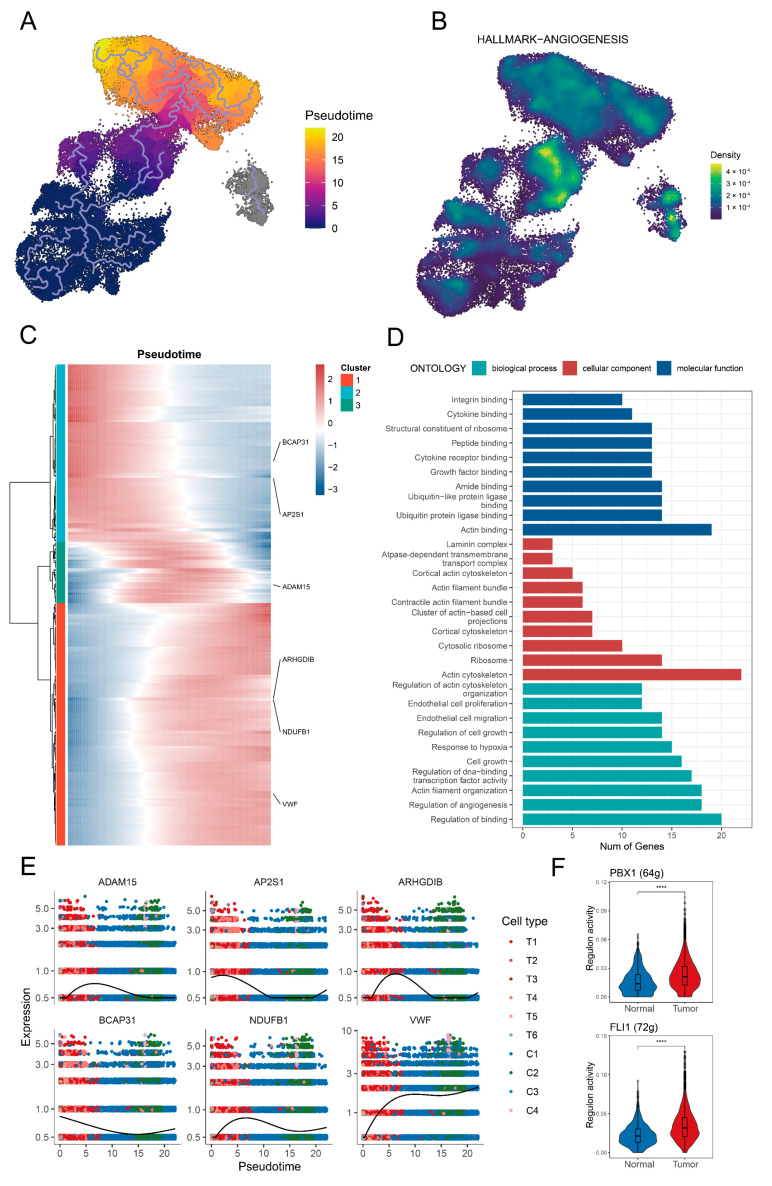
Identification of the transition signature from tip endothelial cells to capillary endothelial cells. (**A**) The pseudotime trajectory illustrates the transition from tip endothelial cells toward capillary endothelial cells. The gradient colour indicates the pseudotime. (**B**) Enrichment level of the angiogenesis pathway in the tip endothelial cells and capillary endothelial cells. (**C**) A heat map shows dynamic shifts in gene expression along the trajectory. The gradient colour reflects expression levels. (**D**) A bar graph displays the GO enrichment results, where the colour denotes distinct pathways and the bar height represents the number of matched genes. (**E**) The scatter plot demonstrates consistent changes in the expression of six key genes (*ADAM15*, *AP2S1*, *ARHGDIB*, *BCAP31*, *NDUFB1*, and *VWF*) across pseudotime. The colours represent different endothelial cell subpopulations. (**F**) A box plot depicts two key differentially active transcription factors in the T1 cluster between normal tissue and tumour tissue, including PBX1 and FLI1. The significance level was tested by the Wilcoxon rank-sum test. Significance levels are denoted as follows: *p* < 0.05, *p* < 0.01; *p* < 0.001, and *p* < 0.0001 (****).

Tip ECs were identified as the root of the cellular differentiation trajectories based on the lower differentiation level (Supplementary Figure S1). Subsequently, a clear cellular trajectory from tip ECs toward capillary ECs was delineated (Figure 2A). TC-ECs along this trajectory showed enhanced activities in oxidative phosphorylation, angiogenesis, and the Wnt/β-catenin pathway (Supplementary Figure S2A and Figure 2B). Key hub genes associated with angiogenesis in TC-ECs included *SLCO2A1*, *PTK2*, *JAG2*, *FSTL1*, and *FGFR1* (Supplementary Figure S2B). Additionally, the identified transitional signature along the trajectory was associated with cellular migration pathways, including actin binding, actin cytoskeleton organisation, and actin filament regulation (Figure 2C,D). Along the cellular trajectory, six critical genes implicated in cell migration, angiogenesis, and vascular function (*ADAM15*, *AP2S1*, *ARHGDIB*, *BCAP31*, *NDUFB1*, and *VWF*) were further identified (Figure 2E). Furthermore, the transcription factors PBX1 and FLI1 exhibited higher activity in the tumour-derived T1 cluster, suggesting their potential regulatory roles in driving these downstream changes (Figure 2F).

### 3.3. Potential Prognostic Significance of Endothelial Transition Signature

**Figure 3:**
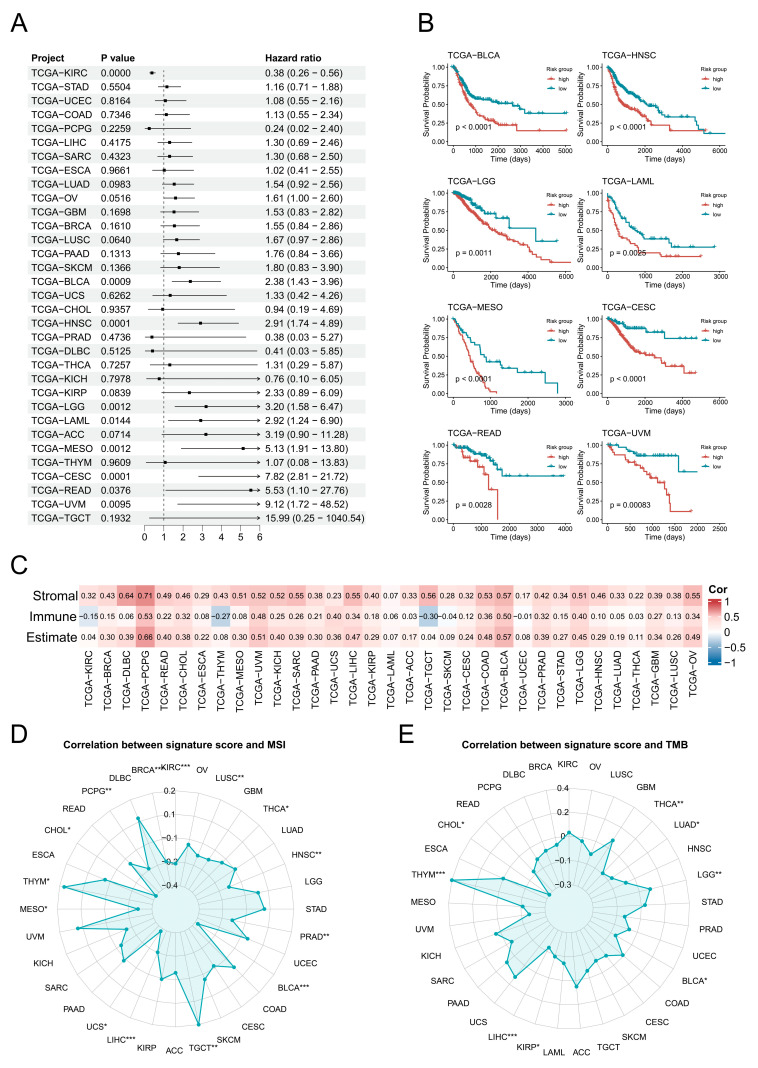
Pan-cancer analysis of endothelial transition signature across cancers. (**A**) A forest plot demonstrates the hazard ratio of the endothelial transition signature across cancers, with bar lengths proportional to the hazard ratio values. (**B**) Kaplan–Meier survival curves reveal significant differences (*p* < 0.05) in overall survival between high-risk and low-risk groups in six cancers, with significance assessed by the log-rank test. (**C**) The correlation heat map displays correlations between the cellular abundance and endothelial transition signature score across cancers. The colour in the matrix represents correlation levels. (**D**,**E**) Radar charts show correlations between MSI (left) and TMB (right) and the endothelial transition signature score. Significance levels are denoted as follows: *p* < 0.05 (*), *p* < 0.01 (**), *p* < 0.001 (***), and *p* < 0.0001.

Further analysis revealed that the EC transition signature score was a significant risk factor (*p* < 0.05) across eight cancer types: BLCA, HNSC, LGG, LAML, MESO, CESC, READ, and UVM (Figure 3A). Subsequent survival analyses validated that higher signature scores were consistently associated with poorer prognosis in these cancers (Figure 3B). Additionally, the EC transition signature score showed positive correlations with the stromal and immune cell abundances in most cancers, except for the immune abundance in KIRC, THYM, and TGCT (Figure 3C). Signature scores exhibited significant positive correlations (*p* < 0.05) with MSI in THYM and TGCT, while they were negatively correlated in 14 cancer types including BLCA (Figure 3D). Furthermore, the EC transition signature scores demonstrated significant positive correlations (*p* < 0.05) with TMB in LGG and THYM but negative correlations in six cancers including BLCA (Figure 3E).

### 3.4. Cellular Communication Pattern in Bladder Cancers

**Figure 4:**
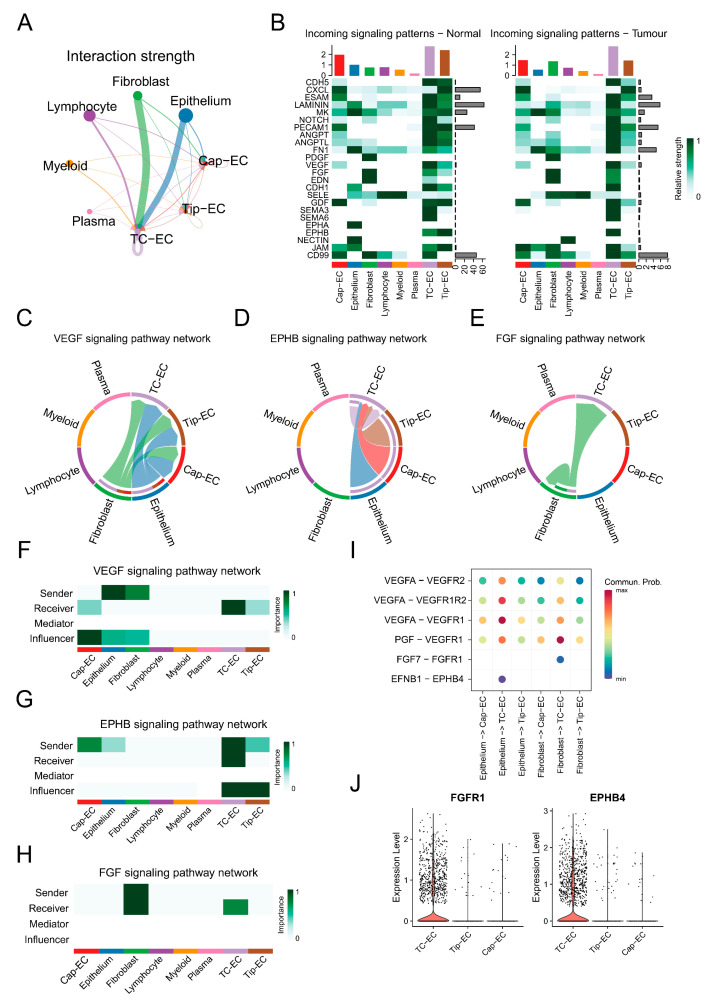
Cell communication patterns in bladder cancer. (**A**) A circle plot demonstrates the overall interaction strength toward endothelial cells from different cells in bladder cancer. The size of the dots shows the overall communication strength in the cells, the arrow direction reflects the signal sender or receiver, and the arrow thickness corresponds to the communication strength. (**B**) A heat map shows communication strengths across different signalling pathways. The colour intensity represents the communication strength, while the column height indicates the summed intensity for each pathway or cell type. (**C**–**E**) Chord diagrams visualise intercellular interactions in the VEGF (left), EPHB (middle), and FGF (right) pathways, where colours denote cell types. The arrow direction reflects the signal sender or receiver, and the arrow thickness corresponds to the communication strength. (**F**–**H**) Heat maps illustrate the functional roles of distinct cell types in the VEGF (top), EPHB (middle), and FGF (bottom) pathways. The role weight is depicted by a gradient colour scale. (**I**) A dot plot reveals the ligand–receptor pairs within the VEGF, EPHB, and FGF pathways. The colour intensity represents the communication strength. (**J**) A violin plot compares the expression of FGFR1 and EPHB4 among multiple cell types, where the y-axis reflects relative expression levels.

Subsequently, bladder cancer was specifically selected for further validation in light of the strong association of prognosis, MSI, TMB, and tissue enrichment of TC-ECs in it. In bladder cancer, TC-ECs exhibited stronger cellular communication compared to capillary ECs and tip ECs, particularly in angiogenesis-related pathways such as FGF, VEGF, and EPHB signalling (Figure 4A,B). TC-ECs received enhanced VEGF signalling from fibroblasts and epithelial cells relative to capillary ECs and tip ECs (Figure 4C). Furthermore, specific interactions involving FGF signalling and EPHB signalling were observed uniquely in TC-ECs (Figure 4D,E). TC-ECs mainly acted as receivers of this signalling, while uniquely serving as senders or influencers in EPHB singling (Figure 4F–H). Six ligand–receptor pairs within these pathways were identified, demonstrating stronger signalling mediated by VEGFA or PGF ligands and distinct interactions mediated by FGF7 or EFNB1 ligands (Figure 4I). These ligand–receptor specificities may arise from the higher expression levels of the *FGFR1* and *EPHB4* receptors in TC-ECs (Figure 4J).

### 3.5. Construction and Evaluation of the Prognostic Prediction Model

**Figure 5:**
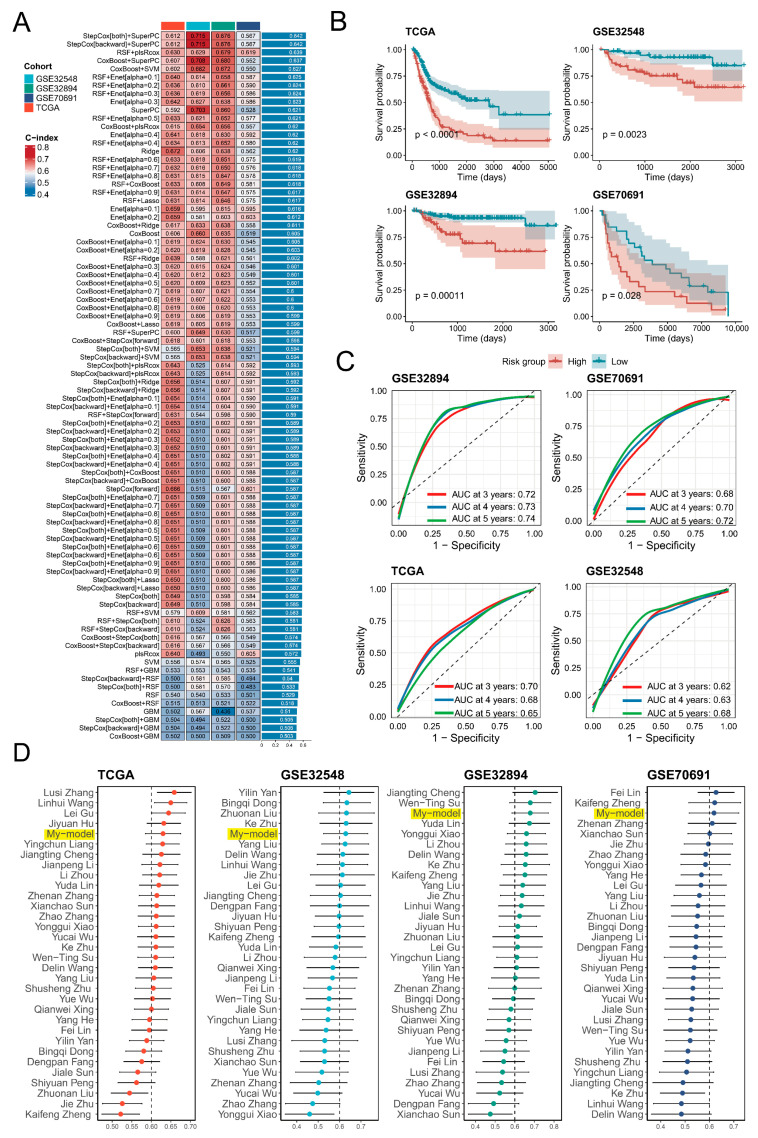
Construction and validation of a prognostic model based on endothelial transition signature. (**A**) A heat map presents the C-index values of various prognostic models generated using multiple machine-learning methods, tested in the training set (TCGA-BLCA) and three independent validations (GSE32548, GSE32894, GSE70691). The colour represents the magnitude of the C-index. (**B**) Kaplan–Meier survival curves reveal significant differences (*p* < 0.05) in overall survival between high-risk and low-risk groups in all cohorts, with significance assessed by the log-rank test. (**C**) Receiver operating characteristic (ROC) analyses assessed the predictive performance at 3, 4, and 5 years in all cohorts. Three colours denote survival in the third year, fourth year, and fifth year, respectively. (**D**) A forest plot demonstrates the C-index values of different models across four independent cohorts (TCGA-BLCA, GSE32548, GSE32894, GSE70691), with bar lengths proportional to the C-index values.

Subsequently, 35 key genes from the endothelial transition signature were selected to construct a prognostic model. Among multiple machine-learning algorithms, the RSF + plsRCox model with 11 genes exhibited the best performance, achieving a C-index of 0.6 in the training set and across three independent validation tests (Figure 5A). Survival analysis revealed significant differences (*p* < 0.05) between the risk groups, and the ROC curves demonstrated satisfactory predictive power, with most AUC values exceeding 0.65 at 3, 4, and 5 years (Figure 5B,C). Compared with 31 published models, the model demonstrated superior prognostic performance with the top five C-index across the TCGA-BLCA, GSE32894, GSE32548, and GSE70691 cohorts (Figure 5D). Additionally, univariate Cox regression analysis confirmed the risk score as a strong prognostic indicator with hazard ratios of 2.95 (Table 2). Finally, protein-level validation of model genes was supported by IHC images from the Human Protein Atlas database (Supplementary Figure S3). The final risk-scoring formula was as follows: Risk Score = 0.165 × *HSPG2* + 0.116 × *HTRA1* + 0.118 × *F2R* + 0.141 × *IDI1* + 0.170 × *RPL21* − 0.116 × *PLXND1* + 0.126 × *PTMS* + 0.096 × *VWA1* − 0.170 × *LAMB1* − 0.262 × *LITAF* + 0.062 × *MALL*.

### 3.6. Characterisation of the High-Risk Group in Terms of Tumour Progression and Cellular Infiltration

**Figure 6:**
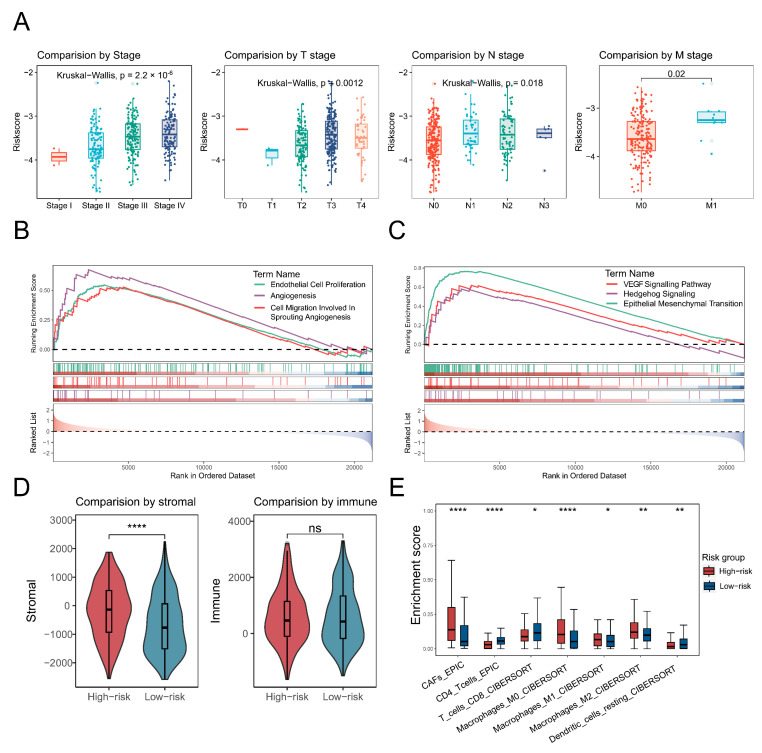
Characterisation of the high-risk group in terms of tumour progression and cellular infiltration. (**A**) A box plot shows the risk scores across different tumour stages, with distinct colours representing each stage. (**B**,**C**) Gene set enrichment analysis (GSEA) illustrates angiogenesis-related (left) and cancer-related pathways. Different colours indicate distinct pathways, and curve directions referred to up- or down-regulation. (**D**,**E**) Box plots depict significant differences (*p* < 0.05) in stromal and immune scores, as well as cellular infiltration, when comparing high-risk and low-risk patients. The significance level was tested by the Wilcoxon rank-sum test. Significance levels are denoted as follows: *p* < 0.05 (*), *p* < 0.01 (**), *p* < 0.001, and *p* < 0.0001 (****).

Significant differences (*p* < 0.05) in risk scores across clinical stages suggested a potential relationship between the risk score and tumour progression (Figure 6A). Angiogenesis-associated pathways, including EC proliferation, migration, and overall angiogenesis, showed elevated activity in the high-risk group (Figure 6B). Additionally, cancer-related signalling pathways such as epithelial–mesenchymal transition, VEGF, and Hedgehog signalling were also upregulated in the high-risk group (Figure 6C). Stromal score by the ESTIMATE algorithm was significantly higher (*p* < 0.0001) in the high-risk group, but there was no significant difference in immune scores (Figure 6D). Furthermore, the high-risk group exhibited an increased infiltration of cancer-associated fibroblasts and macrophages, coupled with a reduced abundance of T cells and dendritic cells (Figure 6E).

### 3.7. Characterisation of High-Risk Patients in Terms of Immunotherapy and Chemotherapy

**Figure 7:**
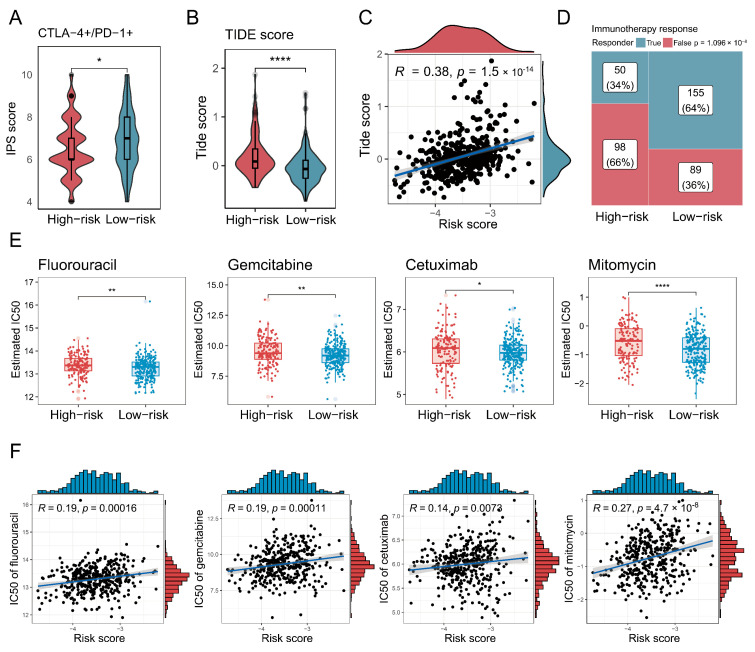
Characterisation of high-risk patients in terms of immunotherapy and chemotherapy. (**A**,**B**) Box plots demonstrate the difference in immunophenotypic scores (IPSs) and TIDE scores between high-risk and low-risk groups. The significance level was tested by the Wilcoxon rank-sum test. (**C**) A scatter plot shows how risk scores correlated with TIDE scores, with Spearman’s correlation used to assess association strength. (**D**) A mosaic plot presents the immunotherapy responses predicted by the TIDE algorithm for high-risk and low-risk groups, where significance was tested via the chi-square test. (**E**) Box plots demonstrate the difference in half-maximal inhibitory concentration (IC50) values between high-risk and low-risk groups. The significance level was tested by the Wilcoxon rank-sum test. (**F**) Scatter plots show how risk scores correlated with IC50 values for various anti-HNSCC drugs, with Spearman’s correlation used to assess association strength. Significance levels are denoted as follows: *p* < 0.05 (*), *p* < 0.01 (**), *p* < 0.001, and *p* < 0.0001 (****).

Finally, the association between the risk score and therapeutic responses or drug sensitivity was evaluated. First, IPS values for CTLA-4 and PD-1 positivity derived from the TCIA database were significantly lower (*p* < 0.05) in the high-risk group (Figure 7A). The TIDE scores by the TIDE algorithm were also higher in the high-risk group, and there was a significantly positive correlation (*p* < 0.001) between the TIDE score and risk score, with a coefficient correlation of 0.38 (Figure 7B,C). Additionally, fewer immunotherapy responders were observed in the high-risk group (Figure 7D). Moreover, IC50 values for several clinically relevant drugs for bladder cancer were higher in the high-risk group and positively correlated with the risk score (Figure 7E,F).

## 4. Discussion

This study identified a novel EC transition signature across multiple cancers, focusing particularly on its prognostic relevance in bladder cancer. The endothelial transition represents the maturation process of newly formed vessels post-angiogenesis, reflecting active angiogenic remodelling. Tumour angiogenesis predominantly occurs through vessels sprouting from pre-existing blood vessels, wherein ECs differentiate into tip cells to guide vessel growth toward angiogenic stimuli [30]. Following contact and fusion with adjacent sprouts, tip cells transition into mature vessels stabilised by pericytes and adhesion molecules, eventually differentiating into mature endothelial phenotypes [31,32]. Consequently, this identified EC transition signature likely reflects angiogenic activity from a distinct perspective, providing novel insights into cancer prognosis and therapeutic interventions. In this study, obvious alterations in the tip and capillary EC populations suggest active cellular transitions between them in tumour tissues.

Pseudotime trajectory analysis further delineated this cellular transition, identifying a critical transitional cluster termed TC-ECs, enriched specifically in tumour tissues and exhibiting enhanced angiogenic activity. This transition signature was notably associated with EC proliferation and migration, aligning closely with known mechanisms of angiogenesis [33]. Within this signature, six key genes were highlighted: *ADAM15*, *AP2S1*, *ARHGDIB*, *BCAP31*, *NDUFB1*, and *VWF*. *ADAM15* promotes endothelial permeability and EC survival, whereas *AP2S1* mediates endocytosis, potentially regulating angiogenic signalling through ligand–receptor internalisation [34,35,36]. *ARHGDIB* modulates cytoskeletal dynamics via Rho family GTPases, potentially influencing tip cell migration [37,38]. *BCAP31*, recognised as a prognostic factor across various cancers, supports angiogenesis via galectin-3 upregulation [39,40]. *NDUFB1* participates in mitochondrial respiratory chain function, suggesting a metabolic dimension to the endothelial transition [41]. Finally, *VWF* synthesised by ECs indicates increasing EC maturity and vascular stability [42]. The transcription factors PBX1 and FLI1, known regulators involved in angiogenesis, were observed to be upregulated in TC-ECs from tumour tissue, further emphasising their roles in endothelial differentiation and vascular remodelling [43,44].

Validation using TCGA data established the EC transition signature as a prognostic indicator across multiple cancers, particularly in bladder cancer. For bladder cancer, there was also a significant correlation (*p* < 0.05) between the EC transition signature score and cellular infiltration, MSI, and TMB. Moreover, TC-ECs, the key cluster in the EC transition, were the most enriched in bladder cancer. Consequently, bladder cancer was specifically selected for further analysis. Cellular communications in this cancer demonstrated that TC-ECs showed notably enhanced interactions via angiogenesis-related signalling pathways, including VEGF, EPHB, and FGF signalling. FGF signalling supports EC proliferation, survival, and vascular stabilisation by FGFR1, whereas EPHB signalling promotes endothelial organisation and vessel maturation by EPHB4 [45,46,47,48]. Elevated VEGF signalling by VEGFA in TC-ECs further underscores its role in promoting angiogenesis and disease progression [49].

To optimise the clinical applicability of this signature, a prognostic model was developed by using multiple machine-learning methods. The ultimate model demonstrated robust predictive capacity across multiple independent cohorts, achieving competitive C-index and ROC curve performance. Elevated risk scores also exhibited advanced cancer stages, heightened activities in angiogenic or cancer-related pathways, and higher stromal infiltration. Interestingly, there were no significant differences in overall immune infiltration between the risk groups, although worse prognosis often indicated less immune infiltration and weaker immune function. T-cell and dendritic cell abundances declined as expected, whereas macrophage levels were significantly elevated (*p* < 0.05). That may be caused by the special involvement of macrophages in angiogenesis, guiding vessel maturation through the secretion of pro-angiogenic factors and physical interactions with ECs [50,51]. Therapeutically, high-risk patients exhibited diminished responsiveness to treatments for bladder cancer, including immunotherapy and several clinical chemotherapy agents [52]. The relationship between the risk score and clinical drug sensitivity highlights the potential of this model in predicting drug responsiveness. This could be used for patient stratification to better guide drug selection. Additionally, this correlation may suggest that the transitional processes identified at the single-cell level could be linked to chemotherapy efficacy, offering new perspectives for improving chemotherapy drugs.

Despite these advances, several limitations should be acknowledged. EC subpopulation analyses were predominantly restricted to tip and capillary ECs, potentially overlooking the contributions of other EC populations to tumour progression. The validation of the transition signature requires further experimental verification. Additionally, the AUC of the ROC curve was around 0.7, and the accuracy was not excellent, although it outperformed other models in the model comparison. Subsequent research should aim to experimentally confirm the mechanistic roles of signature genes and incorporate comprehensive analyses of other cells to provide a more comprehensive understanding, optimising the predictive accuracy of the ultimate model.

In conclusion, this research identified a novel EC transition signature and a transition cluster called TC-ECs, which were characterised by pathway activity, cellular communication, and transcription factor activity. Furthermore, the developed prognostic model effectively stratifies patient risk groups, revealing differences in survival outcomes, tumour progression, cellular infiltration, and treatment responses. Our findings provide a valuable tool for patient stratification and personalised treatment, advancing the understanding and management of bladder cancer.

## 5. Conclusions

This study presents a novel endothelial transition signature that plays a crucial role in the progression of bladder cancer. By integrating single-cell and bulk transcriptome data, we identified a specific endothelial cell subpopulation, tip-to-capillary endothelial cells (TC-ECs), which is enriched in tumour tissues and significantly associated with cancer progression. The transition of endothelial cells from tip to capillary states was found to be strongly linked with angiogenesis, cellular migration, and vascular stability. Additionally, we developed a robust prognostic model based on this endothelial transition signature, which demonstrated superior predictive performance compared to existing models. This model offers valuable insights for stratifying patients based on their risk, improving the understanding of tumour microenvironment interactions, and guiding personalized treatment strategies. Our findings highlight the potential of targeting endothelial cell transitions as a therapeutic approach in bladder cancer and potentially other malignancies, paving the way for more effective and individualized cancer treatments.

## Figures and Tables

**Table 1:** Marker genes for annotation of endothelial cell subpopulations.

Cell Type	Marker Genes	
Tip endothelial cell	*PGF*	*PXDN*
Venous endothelial cell	*ACKR1*	*SELP*
Capillary endothelial cell	*CA4*	*CD36*
Arterial endothelial cell	*FBLN5*	*GJA5*
Lymphatic endothelial cell	*PROX1*	*CCL21*

**Table 2:** Univariate Cox regression of risk groups and clinical features.

Variable	HR	95% CI	*p*
Risk score	2.95	2.10–4.15	4.54 × 10^−10^
Stage	1.70	1.11–2.59	1.49 × 10^−2^
Gender	0.89	0.64–1.24	4.80 × 10^−1^
Age	1.03	1.02–1.05	3.17 × 10^−5^
T stage	1.69	1.23–2.33	1.13 × 10^−3^
Cigarettes per day	1.01	0.97–1.06	6.27 × 10^−1^
M stage	2.96	1.35–6.48	6.72 × 10^−3^
N stage	1.54	1.16–2.03	2.59 × 10^−3^

## Data Availability

The datasets in this study are available from TCGA (https://portal.gdc.cancer.gov/, accessed on 16 January 2024), GEO (https://www.ncbi.nlm.nih.gov/geo/, accessed on 16 January 2024), CellxGene (https://cellxgene.cziscience.com, accessed on 16 January 2024), and the Curated Cancer Cell Atlas (https://www.weizmann.ac.il/sites/3CA/, accessed on 16 January 2024).

## Supplementary Materials

The following supporting information can be downloaded at: https://www.mdpi.com/article/10.3390/biology14050486/s1, Supplementary Figure S1. Cell differentiation level for defining the root of cellular trajectory. (A) The boxplot demonstrates the cell differentiation level across different endothelial subpopulations. (B) UMAP visualisation of cell differentiation level across different endothelial subpopulations. Supplementary Figure S2. Pathway enrichment analysis across different endothelial subpopulations. (A) The dot plot shows the upregulated and downregulated pathways in distinct methods across different endothelial subpopulations. (B) The heat map reveals the correlation in distinct methods between the top 20 hub genes and angiogenesis activity of cell differentiation level across different endothelial subpopulations. The colour intensity represents the correlation level. Supplementary Figure S3. Immunohistochemistry image of signatures in the developed model. Immunohistochemistry images show protein expression levels (high, medium, low, or not detected) for the selected signatures in the developed prognostic model. The used antibodies included HSPG2 (HPA072690), HTRA1 (HPA036655), F2R (CAB008973), IDI1 (HPA039169), RPL21(HPA047252), PLXND1 (CAB020819), PTMS (HPA038186), LAMB1 (HPA004056), LITAF (HPA006960), and MALL (HPA051410). Supplementary Table S1. Samples in scRNA-seq data analysis. Supplementary Table S2. Collected published prognostic models for comparison.

## Abbreviations

The following abbreviations are used in this manuscript:

ECs	Endothelial cells
Enet	Elastic net
GBM	Generalised boosted regression modelling
GSEA	Gene set enrichment analysis
GSVA	Gene set variation analysis
IC50	Half-maximal inhibitory concentration
IHC	Immunohistochemistry
IPS	Immunophenoscore
MSI	Microsatellite instability
PlsRcox	Partial least squares regression for Cox
RSF	Random survival forest
ScRNA-seq	Single-cell RNA sequencing
SuperPC	Supervised principal components
Survival-SVM	Survival support vector machine
TC-ECs	Tip-to-capillary endothelial cells
TIDE	Tumour Immune Dysfunction and Exclusion
TMB	Tumour mutation burden
TME	Tumour microenvironment
