# Supplementary figures and images for: Integration of Single-Cell and Bulk Transcriptome to Reveal an Endothelial Transition Signature Predicting Bladder Cancer Prognosis
Source: Biology (Basel). 2025 Apr 28;14(5):486. doi: 10.3390/biology14050486 (PMC12109300; doi:10.3390/biology14050486)

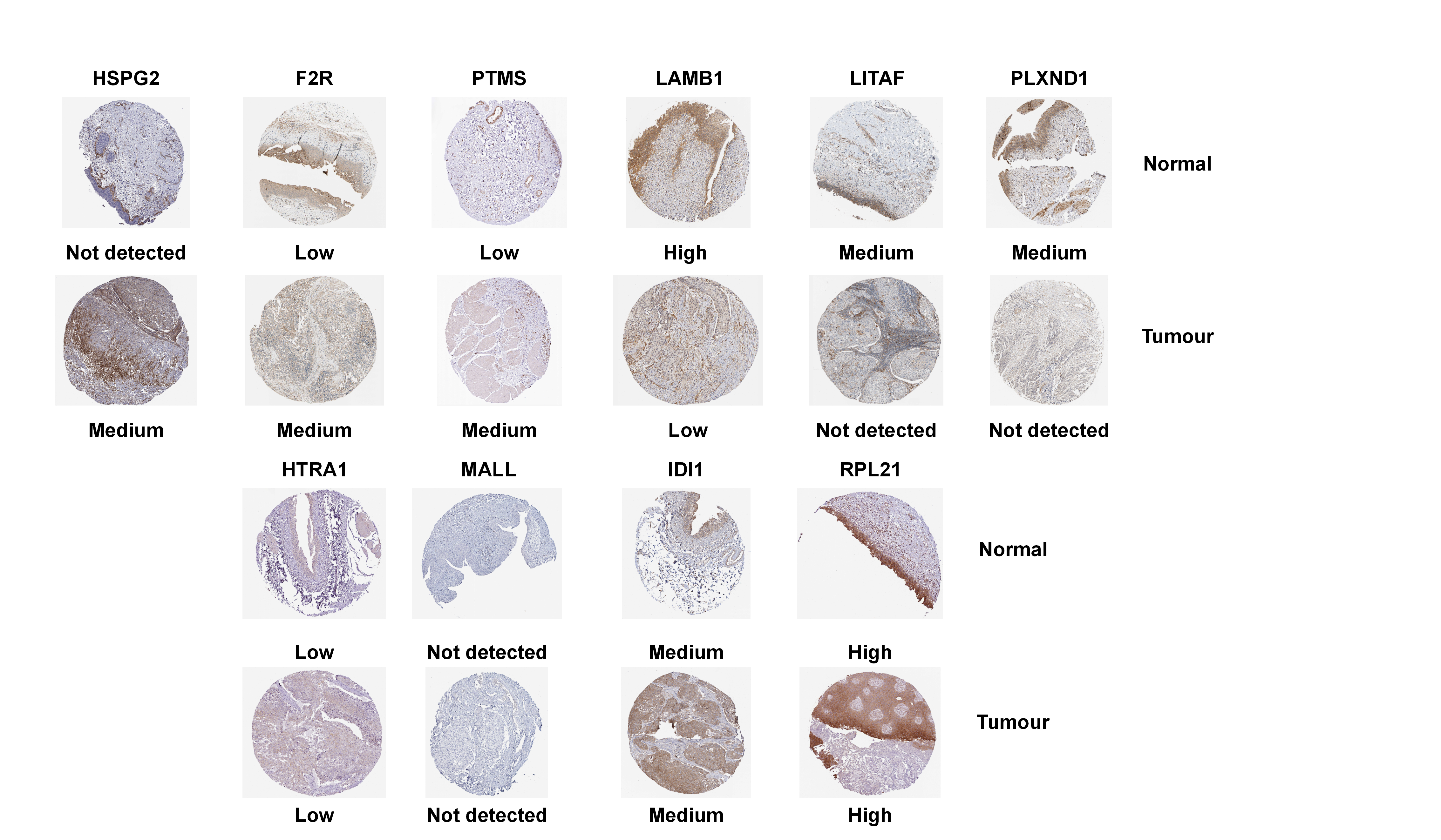

Supplement: Supplementary file 1 [file biology-14-00486-s001.zip › Supplement figure 3.png]
